# Undesired Bulk Oxidation of LiMn_2_O_4_ Increases Overpotential of Electrocatalytic Water Oxidation in Lithium Hydroxide Electrolytes

**DOI:** 10.1002/cphc.201900601

**Published:** 2019-08-13

**Authors:** Max Baumung, Leon Kollenbach, Lifei Xi, Marcel Risch

**Affiliations:** ^1^ Georg-August-Universität Göttingen Institut für Materialphysik Friedrich-Hund-Platz 1 37077 Göttingen Germany; ^2^ Helmholtz-Zentrum Berlin für Materialien und Energie GmbH Nachwuchsgruppe Gestaltung des Sauerstoffentwicklungsmechanismus Hahn-Meitner-Platz 1 14109 Berlin Germany

**Keywords:** aqueous battery, electrocatalysis, oxygen evolution reaction, pH dependence, X-ray absorption spectroscopy

## Abstract

Chemical and structural changes preceding electrocatalysis obfuscate the nature of the active state of electrocatalysts for the oxygen evolution reaction (OER), which calls for model systems to gain systematic insight. We investigated the effect of bulk oxidation on the overpotential of ink‐casted LiMn_2_O_4_ electrodes by a rotating ring‐disk electrode (RRDE) setup and X‐ray absorption spectroscopy (XAS) at the K shell core level of manganese ions (Mn−K edge). The cyclic voltammogram of the RRDE disk shows pronounced redox peaks in lithium hydroxide electrolytes with pH between 12 and 13.5, which we assign to bulk manganese redox based on XAS. The onset of the OER is pH‐dependent on the scale of the reversible hydrogen electrode (RHE) with a Nernst slope of −40(4) mV/pH at −5 μA monitored at the RRDE ring. To connect this trend to catalyst changes, we develop a simple model for delithiation of LiMn_2_O_4_ in LiOH electrolytes, which gives the same Nernst slope of delithiation as our experimental data, i. e., 116(25) mV/pH. From this data, we construct an E^RHE^‐pH diagram that illustrates robustness of LiMn_2_O_4_ against oxidation above pH 13.5 as also verified by XAS. We conclude that manganese oxidation is the origin of the increase of the OER overpotential at pH lower than 14 and also of the pH dependence on the RHE scale. Our work highlights that vulnerability to transition metal redox may lead to increased overpotentials, which is important for the design of stable electrocatalysts.

## Graphical abstract

**LiMn_2_O_4_ oxidizes by delithiation** in LiOH at pH values below 14, as confirmed by X‐ray absorption spectroscopy. This chemical modification reduces the catalytic activity for oxygen evolution and induces a pH dependence on the reversible hydrogen electrode scale. The results indicate that vulnerability to transition‐metal redox reactions may lead to increased overpotentials, which is important for the design of stable electrocatalysts.

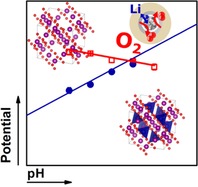

## 1 Introduction

Efficient storage of energy is one of the challenges in turning away from fossil energy vectors towards renewable energies and decelerate global warming.^1^, ^2^ A promising pathway is water splitting for the production of hydrogen as a sustainable energy carrier. Unfortunately, this reaction is kinetically limited by the oxygen evolution reaction (OER),^3^, ^4^ which mandates the use of an efficient catalyst. However, chemical and structural changes before or during electrocatalysis obfuscate the nature of the active state of electrocatalysts for the OER, which calls for model systems to gain systematic insight.

Previously, we studied LiMn_2_O_4_ as a model catalyst for the OER because it shares the structural motif with the active site of natural photosynthesis. We discussed the electrocatalytic mechanism of LiMn_2_O_4_ for the OER in the context of photosynthesis^5^ and determined the product current due to the OER in NaOH,^6^ which hinted at a significant involvement of the bulk to the measured currents as is the case, e. g., in batteries.

Here, the battery aspects of the material are discussed further from the viewpoint of electrocatalysis. LiMn_2_O_4_ was first reported as a positive electrode material for non‐aqueous batteries by Thackeray et al.^7^ and has since been optimized systematically.^8^ Most relevant for electrocatalysis of LiMn_2_O_4_ is its previous use as an active material for aqueous batteries,^9^ mainly in neutral^9^−^15^ electrolytes but also in alkaline electrolytes.^16^−^18^ LiOH has not been used in aqueous Li‐ion batteries, probably because the reversible potential of LiMn_2_O_4_ delithiation under standard conditions is above the thermodynamic potential in alkaline electrolytes, which can be illustrated by aligning the potential scale relative to the Li/Li^+^ redox (“battery scale”) to the scale of the standard hydrogen electrode (SHE).^9^, ^19^ While OER before delithiation prevents operation as an aqueous battery, it is highly desirable for stable operation as a catalyst for oxygen evolution. Moreover, LiOH electrolytes are interesting for in‐depth fundamental studies as the chemical complexity is reduced to the Li−Mn−O−H system, in contrast to all previous electrocatalytic investigations of LiMn_2_O_4_ in KOH^20^ and NaOH^5^, ^6^, ^21^ electrolytes.

In this report, we investigate the oxygen evolution reaction on LiMn_2_O_4_ in LiOH electrolytes with pH between 12 and 14 using rotating ring disk electrodes (RRDE) and X‐ray absorption spectroscopy (XAS). Disks of LiMn_2_O_4_ show pronounced redox peaks below pH 14 in LiOH, which was not previously reported in other hydroxide electrolytes.^5^, ^6^, ^20^, ^21^ The redox peaks are assigned to Mn redox due to (de/–)lithiation of LiMn_2_O_4_. A simple model for the reversible potential of delithiation is derived, which matches the data well. Furthermore, the onset of oxygen evolution is determined using the ring of the RRDE. Finally, we construct an E^RHE^‐pH diagram based on the model and our experimental data. Predictions regarding the oxidation stability are verified using additional ex situ XAS measurements.

## 2 Results and Discussion

**Figure 1:**
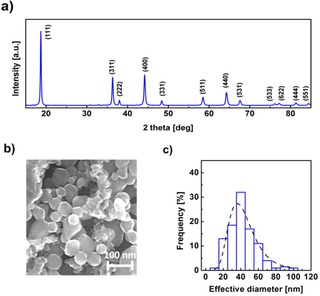
(a) Indexed X‐ray diffractogram of pristine LiMn_2_O_4_ particles (b) SEM image of the nanoparticles and (c) particle distribution (bars) and fitted lognormal distribution (dashed line).

We used the same batch of commercially available LiMn_2_O_4_ nanopowder for our investigations in LiOH as was also used in our previous studies^4^, ^5^ in NaOH where the pristine powder was extensively characterized by scanning electron microscopy (SEM) and transmission electron microscopy (TEM), X‐ray diffraction (XRD) and soft XAS. In short, it has the expected crystal structure of semiconducting LiMn_2_O_4_ (space group *Fd3m*)^5^ with lattice parameter a=8.21(1) Å^4^ that is typical for the composition of Li_1_Mn_2_O_4_.^22^ The size distribution, space group and lattice parameter were confirmed again before the electrocatalytic experiments presented herein and showed similar values as those reported previously, namely a mean diameter 41(15) nm and a median of 40.45 nm (Figure 1). An average manganese valence of +3.5(3) in the bulk was previously determined by soft XAS at the Mn−L edge using calibration to selected experimental references.^5^ The oxide could thus be written Li(Mn^3+^Mn^4+^)O_4_ but we will not make this distinction because XAS can only measure averages. Overall, these analyses demonstrate that the pristine powder has the expected size, bulk crystal structure, bulk composition and bulk valence.

**Figure 2:**
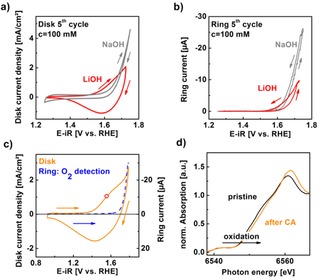
(a) CV of LiMn_2_O_4_ in 100 mM NaOH and LiOH (pH 13) and b) corresponding oxygen detection at ring electrode (detection potential 0.4 V vs. RHE) The NaOH data was taken from ref. [6]. (c) CV of 5^th^ cycle in 10 mM LiOH (solid orange line; pH 12) as well as the corresponding qualitative oxygen detection in LiOH at ring electrode at detection potential 0.4 V vs. RHE (circles) at 1600 rpm rotation. The red bullet indicates 1.55 V vs. RHE. Arrows indicate the scan direction. (d) XANES at the Mn−K edge of a pristine sample and one held at 1.55 V vs. RHE (indicated potential) for 1 h showing an edge shift to higher energies, i. e., oxidation.

The traces of the voltammogram in LiOH evidently differed from our previous studies in NaOH.^4^, ^5^ We performed cyclic voltammetry and simultaneous chronoamperometry at the ring of the used RRDE setup in Ar‐saturated hydroxide electrolytes. Exemplary disk current densities in 100 mM NaOH (gray line) and 100 mM LiOH (red line) are compared in Figure 2a,b during the 5^th^ cycle, which was selected because initial Mn loss had ceased before the 5^th^ cycle in NaOH.^5^ In LiOH, the currents due to Mn loss were higher as compared to NaOH but also changed little after the 5^th^ cycle (Figure S1). Our previous studies showed no evidence of morphological changes of the LiMn_2_O_4_ nanoparticles in NaOH).^5^, ^6^ As the detected Mn loss during the first 5 cycles is similar for all LiOH electrolytes (Figure S1) and also similar to the Mn loss reported in NaOH,^5^ we expect that no changes in morphology occurred in the used LiOH electrolytes. In Figure 2a, the anodic and cathodic traces of the CV in NaOH were featureless except for the exponential rise due to oxygen evolution,^4^, ^5^ while there was a clear anodic shoulder and cathodic peak in the CV in LiOH. The rise in current density due to oxygen evolution occurs at lower voltages in 100 mM NaOH as compared to 100 mM LiOH (Figure 2b), i. e. at a identical pH, which is rationalized below as a chemical change of LiMn_2_O_4_ preceding OER occurring only in 100 mM LiOH.

We investigated the CV of 10 mM LiOH further as it showed a clear exponential rise in current density and emphasized the aforementioned differences to previous studies in NaOH^5^, ^6^ and KOH^20^, ^21^ (Figure 2c). The rising disk current density at high voltage was assigned based on the ring current (blue dashed line), which was set to reduce oxygen at 0.4 V vs. RHE and thus qualitatively detected the oxygen produced at the disk. We only show the anodic trace of the ring currents as the cathodic trace showed hysteresis due to trapped oxygen. As the anodic shoulder and cathodic peak do not show up in the ring current (as expected), it was likely related to manganese redox. Therefore, an additional redox reaction occurred at voltages below the onset of the OER on LiMn_2_O_4_ in 10 mM LiOH as compared to our previous studies in 100 mM NaOH.^5^, ^6^

We gained insight into the manganese redox using hard XAS at the Mn−K edge (Figure 2d). In this spectroscopy, core holes are ejected from the Mn−K shell, which requires a certain threshold energy and produces discontinuities, so‐called “edges”, in the absorption.^23^, ^24^, ^25^ The penetration depth depends on the atomic number and density of the sample.^23^ A single attenuation length is >5 μm for LiMn_2_O_4_ (crystal density 4.3 g/cm^3^)^5^, ^6^ mounted at 45°,^26^ which is clearly a factor 100 larger than the mean particle size. Therefore, the bulk of the particles was probed. The X‐ray absorption near edge structure (XANES) was recorded for the pristine powder (black line) and a sample, which was held at 1.55 V vs. RHE in 10 mM LiOH for one hour (orange line). The spectrum of the latter sample was shifted by 1 eV toward higher energies at a normalized absorption value of 0.5, which is commonly assigned to manganese oxidation. This interpretation is justified because the nuclei of oxidized atoms are less shielded and thus photons of higher energy are needed for excitation of the core hole. A shift of the order of 1 eV corresponds typically to an increase in the average valence of about 0.4 units (i. e. Mn^3.5+^ to Mn^3.9+^).^27^, ^28^, ^29^, ^30^ This is a change in the average bulk valence and can thus only be explained by a bulk process for charge compensation.

We propose that the process associated with Mn oxidation is delithiation of the bulk. This proposal is supported by the use of Li_1‐x_Mn_2_O_4_ as a battery material in similar aqueous electrolytes.^9^, ^10^, ^11^, ^12^, ^14^, ^15^, ^16^, ^18^, ^19^, ^31^, ^32^ The manganese valence can be converted to the lithiation value x using the stoichiometries of the formula Li_1‐x_Mn_2_O_4_, i. e., x is twice the absolute valence change. However, the lithiation of x ≈0.8 estimated from XAS after 1 h at 1.55 V (Figure 2d) likely differed from those at 1.55 V during the CV experiment (Figure 2c) due to the time required for Li diffusion through the bulk of Li_1‐x_Mn_2_O_4_. Nonetheless, the XAS experiment clearly demonstrated that the bulk of LiMn_2_O_4_ can oxidize at voltages where the anodic shoulder was found in the CV. Therefore, we assigned the anodic shoulder in 10 mM LiOH to manganese oxidation and the corresponding cathodic peak to manganese reduction. Charge neutrality was ensured in both cases by lithium extraction or insertion at long time scales. We were not sensitive to changes of the surface. Yet, protonation and deprotonation of the surface likely occurred simultaneously as water is weakly buffering at pH 12 (10 mM LiOH)^33^ and it can thus accept as well as provide protons. For that reason, we expect that the redox properties and the shape of the CV depend on both the lithium concentration and pH (i. e. proton concentration).

In our experiments, both the molarity of Li and the pH changed. Both are intimately coupled in stagnant aqueous solutions as releasing a Li^+^ cation into the electrolyte must be compensated by creation of an OH^−^ anion (assuming water as the only anion source). In our hydrodynamic experiments, they are coupled because the bulk solution contains equal molarities of Li^+^ and OH^−^. The thermodynamic activity is 0.96 in 1.0 M OH and approaches 1 below pH 13.^34^ Therefore, the calculation of the pH from the concentrations is a reasonable approximation. To simplify the discussion, we use the pH defined as

(1) $$pH=14+log([{OH}^{-}])$$

The pH in this report is calculated from the molarity of the prepared LiOH solutions and not measured using conventional glass electrodes as they are inaccurate in LiOH.^34^ We decided to discuss the observed changes in terms of pH changes, which are a staple of systematic electrocatalytic experiments.

**Figure 3:**
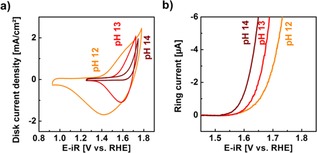
(a) Representative CV during the 5^th^ cycle at pH 12, pH 13 and pH 14 and (b) corresponding qualitative oxygen detection at ring electrode for these concentrations. For all ring electrode measurements, the detection potential was set to 0.4 V vs. RHE at 1600 rpm rotation (complete dataset in Figure S2).

We performed additional RRDE experiments in LiOH electrolytes prepared with pH values between pH 12 (10 mM) and pH 14 (1000 mM) that are shown in Figure 3. The disk scan range was adjusted to clearly evolve oxygen in the anodic range and to completely reduce the particles back to their pristine state (i. e. vanishing current at the end of the cathodic scan). While the same voltage range of 1.25 to 1.75 V vs. RHE was suitable in the range of pH 13 to pH 14, it had to be extended to 0.90 and 1.79 V vs. RHE at the lowest pH 12 (Figure 3a). Moreover, the magnitude and width of the redox peaks clearly depended on the pH where the features were broadest at the lowest pH and vanished at the highest pH. At pH 13, the anodic shoulder appeared to have merged with the onset of currents due to oxygen evolution.

The simultaneously measured ring currents revealed that the onset and kinetics of oxygen evolution likewise depended on the pH (Figure 3b). The ring currents rose exponentially as expected where the lowest onset was found for pH 14 and the highest for pH 12. We defined the onset potential herein as the ring current at −5 μA. The voltage at this reference changes by −40 mV on average when the pH is increased by one unit (Table S1), i. e. a change in the OH^−^ concentration of a factor 10. This shift is the Nernst slope ${\left({\partial E}^{RHE}/\partial pH\right)}_{i=const}$ on the RHE scale. The solubility of O_2_ in hydroxides changes little with concentration in hydroxides,^35^ so that we attribute the observed shifts to modifications of LiMn_2_O_4_, which is also supported by differences in the redox peaks. The most likely chemical modification is a change of the lithiation x in the bulk of ${\mathrm{Li}}_{1-x}{\mathrm{Mn}}_{2}O_{4}$ as also supported by XAS.

Based on the hypothesis of changes in bulk lithiation, we can derive the expected reversible potentials as function of the pH. The derivation is based on earlier work by Li et al.^17^ It is necessary as the plots in literature are derived for a fixed Li molarity^9^, ^19^ (often the standard condition of 1 M Li) which was not the case in our experiments and is often not the case in other recent electrocatalytic studies of pH dependence.^5^, ^36^, ^37^, ^38^, ^39^ The following reaction holds in equilibrium at the pH when Li^+^ will no longer be extracted from LiMn_2_O_4_

(2) $$Li_{1-x}Mn_{2}O_{4}+{x\;H}_{2}O\;↔x\;Li_{aq}^{+}+x\;OH^{-}+x/2{\;H}_{2}$$

We assume that H_2_ forms at the (Pt) counter electrode in its standard state (denoted by superscript 0), i. e. 1 bar. Furthermore, water can be considered in its standard state for the used hydroxide concentrations.^34^ The chemical potentials are thus

(3) $$\mu_{Li}^{LiMn2O4}\left(x\right)+\mu_{H2O}^{0}=\mu_{Li+}+\mu_{{OH}^{-}}+1/2\;\mu_{H2}^{0}$$

The positive and negative charges in the electrolyte must be balanced, i. e.

(4) $$\left(\left[Li^{+}\right]+\left[H^{+}\right]\right)=\left[OH^{-}\right]$$

Since we study basic solutions (i. e. [Li^+^]≫[H^+^]), the proton concentration in Eq. (4) can be neglected. Assuming full dissociation and no interactions, the chemical potentials of the Li and OH in solution can now be obtained from the Nernst equations using the concentrations rather than activities

(5) $$\mu_{Li}=\mu_{Li}^{0}+RT/F\;ln\left(\left[Li^{+}\right]\right)$$

(6) $$\mu_{OH}=\mu_{OH}^{0}+RT/F\;ln\left(\left[OH^{-}\right]\right)$$

Eq. (6) can also be rewritten using the definition of the pH in Eq. (1) as

(7) $$\mu_{OH}=\mu_{OH}^{0}+\ln\left(10\right)RT/F\;\left(pH-14\right)$$

Combining Eq. (3) with Eq. (7) and using that [Li^+^]=[OH^−^], gives

(8) $$\mu_{Li}^{LiMn2O4}\left(x\right)=\frac{2\ln\left(10\right)\;RT}{F}\left(pH-14\right)+\;\mu_{Li+}^{0}+\mu_{OH-}^{0}+\frac{1}{2}\mu_{H2}^{0}-\mu_{H2O}^{0}$$

The voltage in an intercalation battery is given by the difference between the cathode (LiMn_2_O_4_) and anode (Li)

(9) $$E_{cell}=-1/e\;\left(\mu_{Li}^{LiMn2O4}\left(x\right)-\mu_{Li}^{0}\right)$$

where $\mu_{Li}^{0}$ is the chemical potential of lithium metal. The standard chemical potentials correspond to that of the reaction Li+H_2_O ◊ LiOH+0.5 H_2_, for which the free energy is −2.228 eV/e.^17^ At room temperature (T=25 °C), ln(10) RT/F equals 59 mV. Using these values, the voltage of the cell (with a Li/Li^+^ anode) becomes

(10) $$E_{cell}=3.885\;V-0.118\;V\;pH\;$$

The experimental standard potential of LiMn_2_O_4_, $E_{LiMn2O4}^{0}$ , varies to some extend and depends on the synthesis.^12^ Therefore, we obtained it experimentally for our powder in a typical battery electrolyte (Figure S3). The curve shows a first plateau at $E_{LiMn2O4}^{0,low}=3.996$ V vs. Li/Li^+^ (i. e. the anode) for lithiation above x≈0.5 in Li_1‐x_Mn_2_O_4_ and a second plateau $E_{LiMn2O4}^{0,high}=4.138\;\;V$ vs. Li/Li^+^, which correspond to a one‐phase reaction (that of pristine Li_1_Mn_2_O_4_) and a two‐phase reaction.^40^ The half‐cell potential of the LiMn_2_O_4_ cathode is thus

(11) $$E_{LiMn2O4}=3.885\;V\;-E_{LiMn2O4}^{0}-0.118\;V\;pH$$

The values of the pH‐independent term are −0.111 V and −0.253 V for $E_{LiMn2O4}^{0,low}$ and $E_{LiMn2O4}^{0,high}$ . As the duration of delithiation is rather short during a CV at 10 mV/s, we expect that $E_{LiMn2O4}^{0,low}$ is the relevant potential.

This half‐cell voltage is expressed relative to the reversible hydrogen electrode (RHE), as we considered both the standard potential and the pH dependence of the solution in Eq. (7), which is the very definition of the RHE scale. Moreover, Li et al.^17^ have shown experimentally that the cell potential in a conventional non‐aqueous 1 M LiPF_6_ PC/EC electrolyte and 1 M LiOH electrolyte are nearly identical. The half‐cell potential can thus be expressed against the RHE without considering further reactions. Finally, LiMn_2_O_4_ delithiation occurs on the anode in our application and the hydrogen redox on the cathode and so the polarity of the cell must thus be opposite as derived above

(12) $$E_{LiMn2O4}^{RHE}=-0.111\;V+0.118\;V\;pH$$

This equation can now be used to predict whether LiMn_2_O_4_ will delithiate in electrolytes with equal molarities of Li^+^ and OH^−^; delithiation occurs for all applied voltages ${E_{appl}\;>E}_{LiMn2O4}^{RHE}$ .

**Figure 4:**
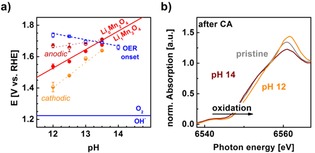
(a) E^RHE^‐pH diagram of Li_1‐x_Mn_2_O_4_ in LiOH showing the expected (solid red line, Eq. 12) and measured (filled circles) reversible potential of delithiation. The half‐filled circles and dotted lines were used in the determination of the experimental value (Table S1). The equilibrium potential of O_2_/OH^−^ (solid blue line) and experimental OER onset determined as the overpotential at −5 μA qualitative ring current (open squares). The dashed line was added as a guide to the eye. Error bars may be too small to be visible. (b) XANES spectra of Mn K‐edge of a LiMn_2_O_4_ electrode holding at 1.55 V vs. RHE at pH 12 and pH 14 compared to pristine LiMn_2_O_4_ powder. Edge shifts to higher energies indicate oxidation of Mn.

We used Eq. (12) and the reversible potential of the OER (1.23 V vs. RHE) to construct an E^RHE^‐pH diagram (Figure 4a). It is related to the more commonly used E^SHE^‐pH (Pourbaix) diagram, that is also used in previous work of aqueous battery work,^9^, ^19^ but the thermodynamic potential of the OER, i. e. the O_2_/OH^−^ equilibrium, on the working electrode (black line) is a horizontal line in the E^RHE^‐pH diagram and the experimental overpotential is also a horizontal line if the Nernst slope vanishes, i. e. ${\left({\partial E}^{RHE}/\partial pH\right)}_{i=const}=0$ , which is the expected pH dependence based on common mechanisms with proton‐coupled electron transfers.^5^, ^36^ Moreover, the representation in an E^RHE^‐pH diagram is often preferable in electrocatalysis as it allows to read overpotentials with respect to the OER more directly from the plot. Even minor changes of the overpotential are clearly visible in this diagram, whereas they are difficult to read from the classical E^SHE^‐pH diagram.

The kinetic data from the CVs in Figs. 3 and S2 was overlaid onto the E^RHE^‐pH diagram to compare to the predicted thermodynamic lines (Figure 4a). The overpotentials of the OER at −5 μA ring current (blue squares) were taken as an approximation of the onset of oxygen evolution. The kinetic data must have slightly higher potentials as compared to the thermodynamic O_2_/OH^−^ equilibrium due to the kinetic barrier. However, the majority of the offset (∼0.4 V) is often attributed to the so‐called scaling relations^32^, ^41^, ^42^ between dependent intermediates. Therefore, the experimental data (blue symbols) are found at higher potentials as the thermodynamic potential (blue line). Furthermore, the experimental data clearly depended on pH on an RHE scale with a Nernst slope of −40(4) mV/pH, which clearly differed from our previous study in NaOH where the Nernst slope was −2(1) mV/pH the onset of the OER.^5^

The reversible potential of delithiation of LiMn_2_O_4_ was also determined experimentally. For this, we determined the peak potentials as the zero‐crossings of the second derivative of the measured data (Figure S4). The analysis was performed separately for the anodic and cathodic shoulder/peaks and their average potentials are shown in Figure 3a between pH 12 and 13.5. for higher pH values, no shoulders or peaks were detected. We had assigned the anodic shoulder to Mn oxidation above +3.5 and the cathodic peak to Mn reduction back to about +3.5. The difference between the anodic and cathodic potentials decreases from 260 mV at pH 12 to 71 mV at pH 13.5 in the resistance‐corrected data. Thus, we attribute the diminishing difference in shoulder/peak potentials to better reversibility^43^ of lithium extraction and re‐insertion at higher LiOH concentrations, i. e. closer to standard conditions. The reversible potential was determined as the midpoint potentials of the anodic and cathodic shoulders/peaks (red symbols), i. e. their average. The determined midpoint potentials fall onto the line predicted by Eq. (12) within error, except for pH 13.5 because it was too close to the intersection of the OER onset (see below). Thus, we confirmed that the assumptions and simplifications made in the derivation are valid and reasonable for our application.

Our E^RHE^‐pH diagram can be used to predict whether LiMn_2_O_4_ will retain its bulk valence of +3.5 during electrolysis in LiOH electrolytes, which is important for the stability and activity of the electrocatalyst. The fit line to the experimental oxygen evolution data of the ring electrode intersects that of the (de/–)lithiation reaction (Eq. 12) slightly below pH 13.5. Had we read the voltages at a lower ring current, then the intersection would occur at a slightly lower pH value. This intersection is highly significant because oxygen evolution (blue symbols and line) should be performed at potentials where Li cannot be extracted from LiMn_2_O_4_ (red symbols and lines). Otherwise, the Mn on the surface and in the bulk of LiMn_2_O_4_ oxidize. The E^RHE^‐pH diagram suggests that measurements at pH 12 in LiOH lead to significant Mn oxidation (as discussed above), while those at pH 14 should retain their valence.

The expected bulk valences were again investigated by hard XAS at the Mn−K edge (Figure 4b). A dense pellet of optimized loading was prepared and measured in transmission mode (grey line). It showed a pre‐edge at 6540 eV, a shoulder at 6550 eV and maximum (white line) at 6560 eV. The shape of a XANES spectrum depends on the local geometric and electronic structures of the absorbing atom.^44^ It is not straightforward to interpret and requires extensive theoretical calculations that are beyond the scope of this manuscript. Here, we only compare spectral differences among the samples. The measurements of the LiMn_2_O_4_ electrodes had to be made in fluorescence yield (FY) mode due to the comparably low loading required by the electrocatalytic measurements. The FY mode only approximates an X‐ray absorption spectrum^23^, ^45^ and depends on the measurement geometry. Nonetheless, the spectrum of a sample held at 1.55 V vs. RHE for 1 h at pH 14 is congruent with the pristine sample and only deviates at the maximum near 6560 eV, where lower amplitudes are a common artefact of FY measurements.^46^, ^47^ Thus, we conclude that the bulk material was not changed at 1.55 V in LiOH at pH 14 and in particular retained an average valence of +3.5. In contrast, the sample held at the same potential at pH 12 differed clearly, namely, the shoulder was less pronounced, the edge shifted to higher energies and the maximum clearly increased. Overall, the XAS measurements confirmed our predictions from the E^RHE^‐pH diagram. In particular, the LiMn_2_O_4_ sample measured at pH 14 retained its bulk valence.

The Mn valence has been correlated with the activity for the OER previously.^28^, ^48^, ^49^, ^50^, ^51^ In a more detailed picture, the (bulk) e_g_ occupancy has been proposed to correlate with activity^52^ where an occupancy near unity results in the lowest overpotential. The rationale is that the electron density of the e_g_ orbital points towards the absorbing oxygen, which is a key step in the mechanism.^36^, ^41^ For spinels, including LiMn_2_O_4_ and several closely related spinels, the (bulk) e_g_ occupancy of the octahedral site has been proposed to correlate with the overpotential.^20^ In the simple crystal field splitting model of manganese oxides with valences between +2 and +4, they contain 3 (spin up) electrons in the t_2g_ orbitals and the e_g_ orbital fills up (with spin up) electrons from 0 (Mn^4+^) to 1 (Mn^3+^) to 2 (Mn^2+^).^53^ Based on the previous studies of the e_g_ occupancy, Mn^3+^ (e_g_ occupancy of 1) is desirable in an electrocatalyst for the OER. However, a single parameter does not fully describe the catalytic properties of a material as complex as an oxide.^41^, ^52^, ^54^, ^55^, ^56^, ^57^ Mixed manganese valences between Mn^3+^ and Mn^4+^ result in the highest activity, i. e. lowest overpotential, in many reports.^48^, ^54^, ^58^, ^59^, ^60^, ^61^, ^62^, ^63^, ^64^, ^65^ Therefore, retaining the Mn^3.5+^ valence of the pristine LiMn_2_O_4_ is crucial for sustained electrolysis with unchanged activity.

The observed increase of the OER overpotential from pH 14 to pH 12 in LiOH can now be explained in the context of changed manganese valence. The available literature data suggests that oxides with Mn valences slightly above Mn^3+^ have higher activity than those with lower or higher manganese valence.^20^, ^52^ We have calculated the conditions, for which Mn reduction is expected (Figure 4a) and verified it experimentally for selected points (Figure 4b). Therefore, we conclude that the overpotential increased in electrolytes below pH 14 due to bulk delithiation, which oxidizes the Mn in the bulk of LiMn_2_O_4_ above average valences of +3.5.

It is often assumed, especially in theoretical work,^66^ that the overpotential does not depend on pH, which is sometimes not the case in experimental studies.^36^, ^67^ The origin of this effect is not well understood. While there are probably also other triggers of the effect, our work clearly demonstrates that changes in the bulk valence can induce pH dependency on the RHE scale, i. e. non‐Nernstian behavior, as witnessed by a non‐zero Nernst slope (${\partial E}^{RHE}/\partial pH$ ). The same pH dependence was reported for materials that (de/–)intercalate oxygen.^37^ While few currently studied electrocatalysts will intercalate or deintercalated ions in the voltage range and electrolyte composition where the OER is studied, the classical E^SHE^‐pH (Pourbaix) diagram predicts valence changes for many used oxides, e. g. simple manganese oxides^68^, ^69^ and they were also observed experimentally by in situ XAS.^70^, ^71^, ^72^, ^73^, ^74^, ^75^, ^76^ It is thus likely that many oxides, particularly manganese oxides, show non‐zero Nernst potentials when they are oxidized or reduced in the investigated electrolytes.

## 3 Conclusions

We investigated the effect of bulk oxidation on the overpotential of LiMn_2_O_4_ as an electrocatalyst for the OER in LiOH electrolytes with pH between 12 and 14. We found pronounced redox peaks in LiOH electrolytes with pH≤13.5 that were not observed at pH 14 and in all previous reports where NaOH and KOH electrolytes were used.^5^, ^6^, ^20^, ^21^ Using XAS at the Mn−K edge, we showed that Mn in the bulk of LiMn_2_O_4_ oxidized at pH 12, which we used to assigned the observed peaks and shoulders to the Mn^3.5+^‐Mn^3.5+δ^ redox. As the XAS measurement was bulk sensitive, we argued that the most likely process of charge compensation was delithiation of the bulk. The current due to oxygen evolution could not be determined from the total disk currents due to interference of these Mn redox peaks. Yet, the ring of the used RRDE setup qualitatively measured the onset of oxygen evolution, which occurred at voltages higher than that of the Mn redox. The onset of the OER was pH‐dependent on the RHE scale with a Nernst slope of −40 mV/pH at −5 μA (uncalibrated) ring current. We derived a simple model for the expected reversible potentials of delithiation for the used electrolytes with equal molarities of Li^+^ and OH^−^. The calculation of the expected delithiation potentials needs the standard potential of LiMn_2_O_4_ delithiation, which we determined experimentally in a common battery electrolyte as 3.996 V vs. Li/Li^+^ below x=0.4 in Li_1‐x_Mn_2_O_4_. The predicted Nernst slope of delithiation of 118 mV/pH was identical to the experimental Nernst slope of 116(25) mV/pH within error. The model and experimental data of both delithiation and oxygen evolution were used to construct an E^RHE^‐pH diagram. The lines given by the Nernst slopes of the onset of the OER and that of delithiation intersect near pH 13.5. The E^RHE^‐pH diagram illustrates that delitihation occurs at voltages below that of the onset of OER at pH below about 13.5 while the onset of OER has a lower onset at higher pH. This is significant because the average bulk valance of Mn^3.5+^ will only be retained at pH above about pH 13.5, which we verified experimentally using XAS on a LiMn_2_O_4_ electrode operated at pH 14. Overall, the model and experimental data strongly support bulk delithiation of LiMn_2_O_4_ below a pH of about 13.5. We discussed Mn oxidation due to delithiation in the context of the e_g_ orbital descriptor, where oxidation above Mn^3.5+^ should increase the overpotential for the OER. Therefore, we concluded that bulk delithiation and the concomitant oxidation of Mn^3.5+^ to Mn^3.5+δ^ increased the overpotential of the OER and were the origin of the pH dependence on the RHE scale. The E^RHE^‐pH diagram in our work provides an intuitive graphical tool to gauge the stability of electrocatalyst against redox changes when they are not measured under standard conditions, i. e. 1 M LiOH. While it is most straightforwardly extended to predict the pH dependence of other electrocatalysts that are also common battery materials, e. g., LiCo_1‐x_M_x_O_2_ and LiCoPO_4_,^77^, ^78^, ^79^, ^80^, ^81^ oxidation due to structural changes should also show pH dependence on the RHE scale, e. g., Risch et al.^82^ showed that the Nernst slope of the Co^2/3+^ redox couple differs from that of the Co^3/4+^ redox couple. Therefore, pH dependence on the RHE scale (i. e. non‐Nernstian behavior) should be expected when the electrocatalyst is not stable against redox changes in the investigated electrolyte.

## Experimental Section

### Materials

LiMn_2_O_4_ (>99 %) catalyst powder was purchased from Sigma‐Aldrich. Tetrahydrofuran (THF) was purchased from VWR (≥99.9 % stabilized). For the electrolyte Lithium hydroxide powder (99 %) purchased from Merck was dissolved in ultrapure water (Milli‐Q R≥18.2 MΩ). Argon (5.0) to purge the electrolyte was purchased from AirLiquide Alphagaz. Acetylene carbon black was purchased from Alfa Aesar and was acid‐treated.^83^ All other chemicals were used as received.

### Characterization

The pristine powder was also characterized by a XRD Bruker D8 Discovery with monochromatized Cu‐K_α_ radiation in a two theta range of 15°–85° in 0.05° steps. For this, the LiMn_2_O_4_ powder was glued using rubber cement (Fixo Gum, Marabu GmbH) on a microscope slide made of glass. The particle distribution was determined by Nova Nano SEM 650 in high vacuum mode at 15 kV. Both results were in good agreement with our previous publications^4^, ^5^ and are shown in the supporting information.

### Electrochemical Setup

For our electrochemical experiments we were using an OrigaFlex (OrigaLys SAS) system of three OGF500 potentiostats in bipotentiostat configuration. The RRDE‐setup was composed of an RRDE‐3 A rotator (ALS Japan Co Ltd.) and a custom‐made cylindrical PTFE cell that was used in a three‐electrode configuration, consisting of a saturated calomel electrode (RE‐2B, ALS Japan Co Ltd.) and a platinum counter electrode, which were arrange radially around the working electrodes. The distance between the RRDE‐electrode and counter and reference electrode was 17 mm. We used a RRDE‐electrode made by ALS Japan Co Ltd containing a removable glassy carbon electrode 4 mm in diameter (area 0.126 cm^2^) and a concentric platinum ring electrode with 5 mm inner and 7 mm outer diameter separated by a Teflon spacer. Both working electrodes were separately polished to a mirror finish with Al_2_O_3_‐polish on separate polishing pads and cleaned afterwards with isopropanol. After this cleaning procedure the RRDE was assembled from both parts. This procedure reduces a possible contamination of the electrodes. A RHE (Hydroflex, Gaskatel GmbH) was used to calibrate the SCE to RHE scale.^6^ For XAS measurements we used graphite foil (Alfa Aesar 99.8 %) as the electrode instead of a glassy carbon in the RRDE‐setup.

### Electrochemical Experiments

For catalytic experiments we realized a loading of 50 μg active material on a glassy carbon electrode by drop coating. For this, we applied 10 μL of an ink containing of LiMn_2_O_4_ (83 % of solid part) and carbon black (17 % of solid part) in THF. The Teflon spacer of the RRDE assembly prevents the ink from contacting the ring electrode. If it happened by accident, the electrode was discarded. The electrolyte was saturated with argon gas. For RRDE‐measurements, we used a rotation speed of 1600 rpm. The detection potential for the detection of oxygen was determined in our previous work.^6^ Our protocol includes an impedance measurement from 100 KHz to 1 Hz at the open circuit potential. The ohmic resistance for the iR correction was obtained from this measurement at high frequency where the phase angle approached zero. Additional detail on the used protocol may be found in the supporting information.

### Sample Preparation for Post‐Mortem Characterization by XAS

For XAS measurements the catalytic ink was applied on a graphite foil and a CA at 1.55 V vs. RHE was performed for one hour in a LiOH electrolyte with pH 12 or pH 14. Afterwards the electrode was washed off with Milli‐Q water, dried and transferred to the beamline. To characterize the pristine powder by XAS, we diluted the powder to 1‐wt % of Mn in LiMn_2_O_4_ using BN. After these powders have been homogenized by a mortar and pestle, a pellet of 10 mm in diameter was pressed at 20 bar. This pellet was transferred to the beamline.

### XAS Measurements

XAS measurements of post mortem samples were performed in fluorescence mode (detector: Bruker X‐Flash 6|60) at the KMC‐2 beamline of the BESSY II synchrotron in Berlin. The beamline energy resolution is 1/4000. The acquisition time for one EXAFS scan takes around 90 min. The used beam size was around 2 mm×4 mm (hor. x vert.). The first inflection point in the XANES of a manganese foil was used for the energy calibration by setting it to 6539 eV. XAS measurements of the pristine powder were performed in transmission mode at CLÆSS beamline of ALBA synchrotron in Barcelona. The used beam size was around 1 mm×1 mm. We also used a manganese foil for energy calibration. All XANES spectra were normalized by subtraction of a straight line before the edge and division of a polynomial after the edge.

## Conflict of interest

The authors declare no conflict of interest.

## Supporting information

As a service to our authors and readers, this journal provides supporting information supplied by the authors. Such materials are peer reviewed and may be re‐organized for online delivery, but are not copy‐edited or typeset. Technical support issues arising from supporting information (other than missing files) should be addressed to the authors.

Supplementary
