# Supplementary material for: Undesired Bulk Oxidation of LiMn2O4 Increases Overpotential of Electrocatalytic Water Oxidation in Lithium Hydroxide Electrolytes
Source: Chemphyschem. 2019 Aug 13;20(22):2981–8. doi: 10.1002/cphc.201900601 (PMC6899966; doi:10.1002/cphc.201900601)
Supplement: Supplementary file 1 — Supplementary [file CPHC-20-2981-s001.pdf]

CHEMPHYSCHEM

Supporting Information

© Copyright Wiley-VCH Verlag GmbH & Co. KGaA, 69451 Weinheim, 2019

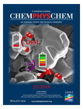

Undesired Bulk Oxidation of $LiMn_2O_4$ Increases Overpotential of Electrocatalytic Water Oxidation in Lithium Hydroxide Electrolytes

Max Baumung, Leon Kollenbach, Lifei Xi, and Marcel Risch*© 2019 The Authors. Published by Wiley-VCH Verlag GmbH & Co. KGaA.
This is an open access article under the terms of the Creative Commons Attribution License, which permits use, distribution and reproduction in any medium, provided the original work is properly cited.An invited contribution to a Special Issue on Electrocatalysis

Electrochemical protocol

1) Ar purge at OCP	15 min
2) Disk EIS	100 kHz – 1 Hz; OCP
3a) Ring CA: O2 Detection	hold 0.4 V vs. RHE until Disk CV finishes
3a) Ring CA: Mn Detection	hold 1.2 V vs. RHE until Disk CV finishes
3b) Disk CV: OER (pH 12)	hold 0.90 V for 5 min, then 0.9 – 1.79 vs. RHE; 10 mV/s; 10 cycles
3b) Disk CV: OER (all others)	hold 1.25 V for 5 min, then 1.25 – 1.75 vs. RHE; 10 mV/s; 10 cycles
4) Disk OCP: Conditioning	300 Sec
5) Disk EIS	100 kHz – 1 Hz, OCP

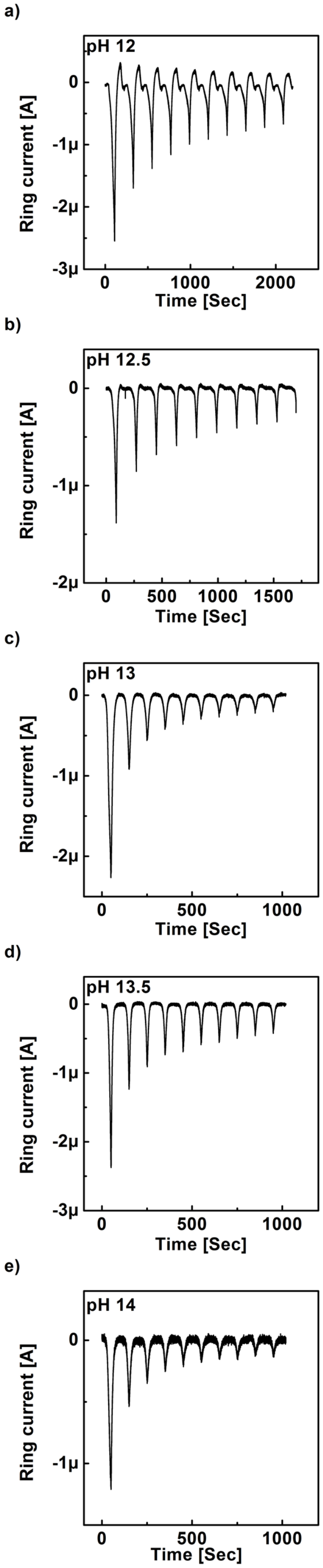

Figure S1. Representative ring currents to detect Mn-loss during cycling for all pH. The ring was set to 1.2 V vs. RHE and rotated at 1600 rpm.

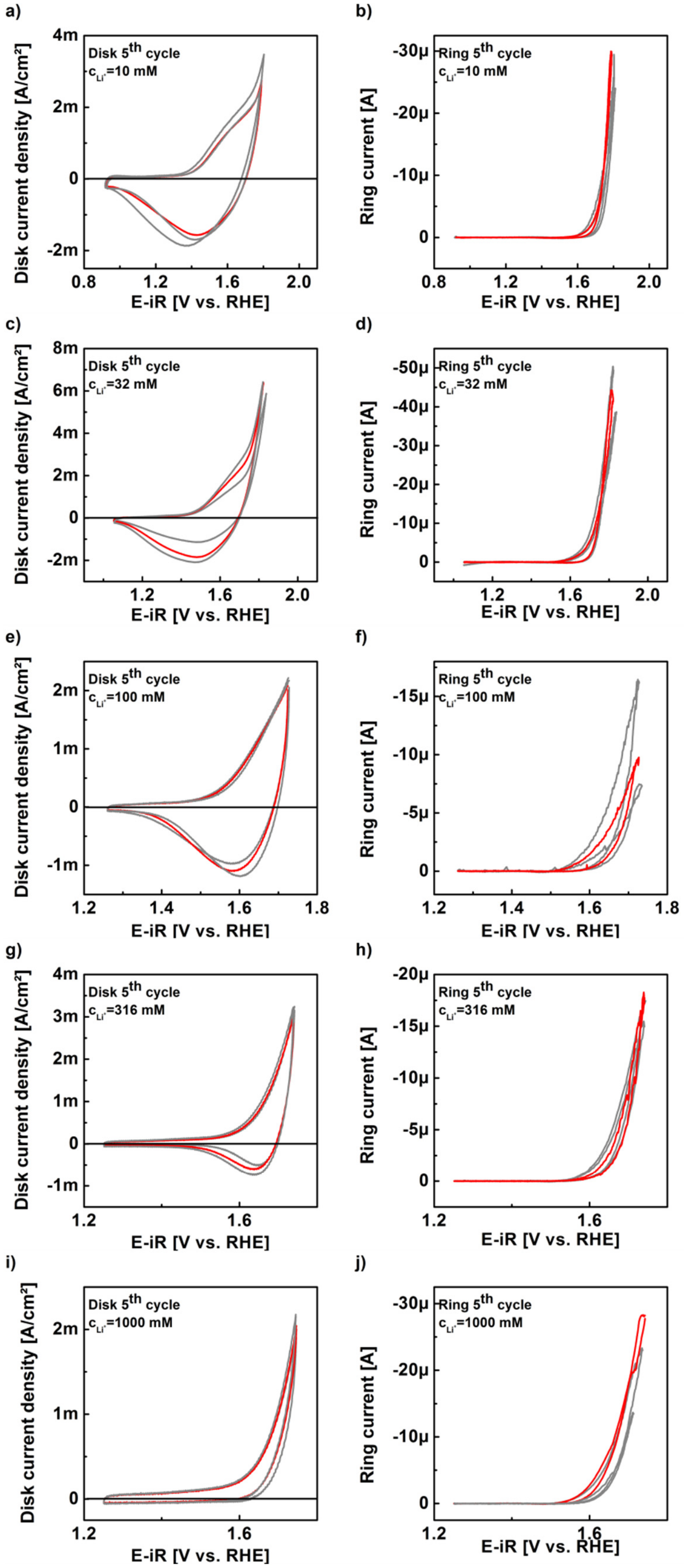

Figure S2. (a,c,e,g,i) CV at disk electrode and (b,d,f,h,j) corresponding ring at each lithium concentration (detection potential 0.4 V vs. RHE; 1600 rpm rotation). The representative CVs selected for the main text are shown in red.

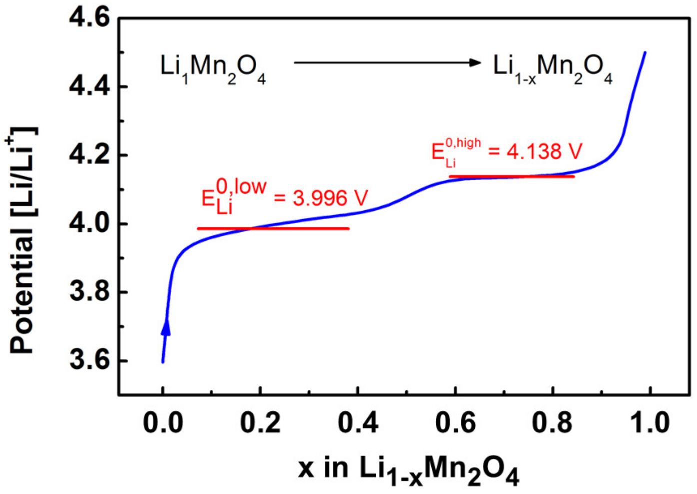

Figure S3. Delithiation of $Li_1Mn_2O_4$ towards $Li_0Mn_2O_4$ in 1 M $LiPF_6$ EC/DMC (50:50) electrolyte using C/12-rate. The cathode contained 83% active material and 17% carbon black. The anode and reference electrode were Li metal. Measurements were performed in a glovebox with < 1 ppm O_2 and water.

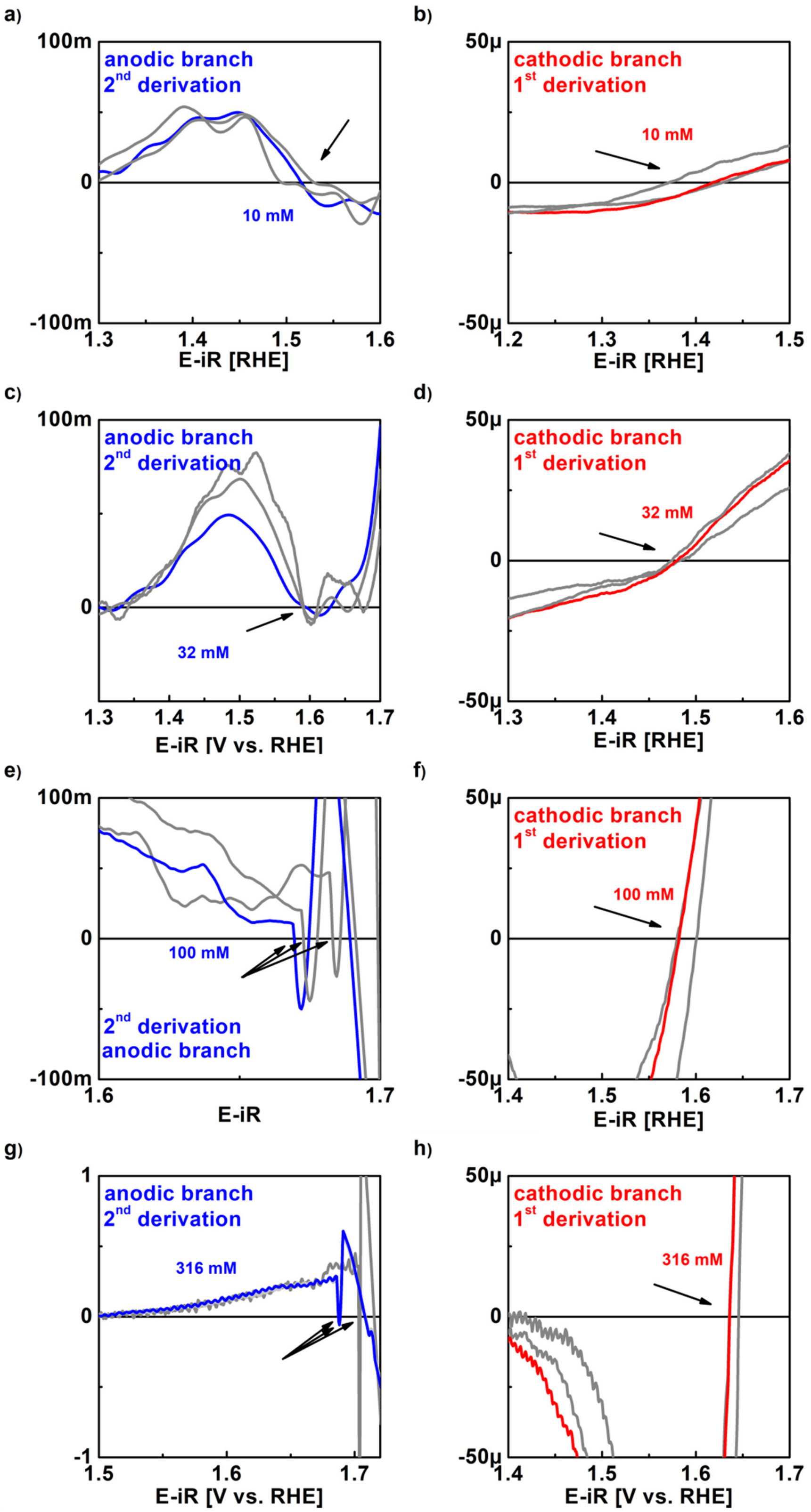

Figure S4. Second derivatives (a,c,e,g,i) of the anodic branch of the CV and (b,d,f,h,j) of the cathodic branch to determine the positions of shoulders and peaks in the CV.

Table S1. Key voltages (in V vs. RHE) of the E^{RHE}-pH diagram in Fig 3a

pH	anodic	cathodic	midpoint	OER onset at -5 µA (ring)
12	1.66(1)	1.40(3)	1.53(1)	1.73(1)
12.5	1.65(5)	1.479(5)	1.568(3)	1.729(3)
13	1.680(7)	1.58(1)	1.633 (6)	1.69(1)
13.5	1.709(3)	1.638(5)	1.673(3)	1.689(5)
14	-	-	-	1.65(1)
Nernst slope	12(9) mV/pH[a]	200(20) mV/pH[a]	116(25) mV/pH[a]	-40(4) mV/pH[b]

[a] determined between and including pH 12 to pH 13

[b] determined between and including pH 12 to pH 14
